# Can Generative Artificial Intelligence Reliably Score Open-Ended Question Assessments in Undergraduate Medical Education?

**DOI:** 10.1007/s40670-026-02638-2

**Published:** 2026-03-03

**Authors:** Doreen M. Olvet, Marieke Kruidering, Tracy B. Fulton, Bao Truong, Kumiko Endo, Robert Lucito, Joanne M. Willey

**Affiliations:** 1 Northwell, New Hyde Park, NY US; 2 Department of Science Education, The Donald and Barbara Zucker School of Medicine at Hofstra/Northwell, Uniondale, NY US; 3 Department of Cellular and Molecular Pharmacology, University of California at San Francisco School of Medicine, San Francisco, CA US; 4 Department of Biochemistry and Biophysics, University of California at San Francisco School of Medicine, San Francisco, CA US; 5 Department of Pediatrics, University of California at San Francisco School of Medicine, San Francisco, CA US; 6 Med2Lab, Inc, 44 Montgomery St. Ste 300, San Francisco, 94104 CA US

**Keywords:** Open-ended question, Generative artificial intelligence, GPT-4, Assessment, Undergraduate medical education

## Abstract

There are numerous benefits to including open-ended questions (OEQs) in the assessment of medical knowledge, but one of the biggest challenges is the time it takes to grade student responses. With the widescale introduction of generative artificial intelligence (AI), it is plausible that OEQ exams can be automatically scored. The purpose of this study was to establish the accuracy of generative AI when scoring medical student OEQ exams. Students’ responses from OEQs administered at two US allopathic medical schools were analyzed. Case vignettes, questions, rubrics, and student responses were fed into the GPT-4 model via the Med2Lab platform. The Med2Lab system was specifically engineered to manage rubric integration and automate prompt workflows. Scores and feedback on students’ responses were generated and compared to faculty scores using Cohen’s weighted kappa (k_w_) to evaluate inter-rater reliability (IRR). An error pattern analysis was performed to assess why there were scoring discrepancies between faculty and GPT-4, then this information was used to perform rubric engineering. We ran 3 iterations of GPT-4 scoring after each rubric adjustment. By the third iteration, IRR between faculty and GPT-4 was substantial using the analytic rubric (question 1A: k_w_=0.94; question 2A: k_w_=0.88) and the holistic rubric (question 2H: k_w_=0.89). IRR for question 1H reached moderate reliability (k_w_=0.54). We identified errors in GPT-4 and faculty scoring, although score discrepancies were typically only 1-point. Our data suggest that generative AI can be used to reliably score OEQ exams using an iterative process of rubric engineering to achieve maximum reliability.

**Supplementary Information:**

The online version contains supplementary material available at 10.1007/s40670-026-02638-2.

## Background

The recent advent of generative artificial intelligence (AI) products has ushered in a new era in education with the potential to revolutionize the way we teach and assess students in the health professions [1, 2]. Generative AI is a large language model that can create new content based on training that uses resources on the internet and elsewhere, as well as user prompts and feedback. It is already being used in the clinical setting as a diagnostic tool [3, 4] and to enhance patient-centered care [5]. Medical educators have been exploring the value of generative AI to develop multiple choice questions [6, 7].

Generative AI can also aid in the assessment of medical students using open-ended questions (OEQs). OEQ exams are beneficial to student learning because they model clinical reasoning [8–10], require learners to communicate their thought process [9, 11], compel learners to alter study habits which stimulate deep learning [12, 13], and reinforce the retention of material through retrieval practice [11, 14]. However, currently only 51% of accredited US allopathic medical schools use OEQs for assessment of medical knowledge [15]. Faculty report that the biggest barrier to including OEQs on exams in medical education is the amount of time it takes to grade student responses [8–10]. Another major concern is that grading can be subjective [8–10, 16]. These concerns prevent medical educators from exploring assessment options beyond multiple choice questions regardless of the benefits of using OEQs.

We have shown that it is feasible to include OEQs in medical school assessment [17], but institutional support and faculty buy-in are crucial for providing the protected time required for grading exams [17, 18]. Without such support, it may not be possible for many institutions to implement OEQs. However, if generative AI can be trained to accurately grade student responses to OEQs, major barriers linked to OEQs would be eliminated. Furthermore, there is often limited feedback to students on their exam performance. Generative AI can provide students with individualized narrative feedback for each response, which has the potential to improve future learning [18].

Assessment practices should be grounded in validity and reliability frameworks to ensure the accuracy of the assessment scores. Both Messick [19] and Kane’s [20] validity frameworks emphasize inter-rater reliability as an essential component to assessment validity and generalization. Prior to the emergence of generative AI, natural language and machine learning methods were used to evaluate the reliability of automated scoring with mixed results. There is high reliability reported when the scoring rubric is binary (i.e., 0 or 1 point) [21, 22], however reliability declines for multi-point rubrics [22–24]. Some research has begun to explore the use of generative AI to grade OEQs in dental and medical education, showing variable correlations between faculty and AI [25–27], however a more robust analysis of inter-rater reliability is needed. The objective of the current study was to establish the accuracy of generative AI when scoring OEQ exam questions compared to faculty content experts. We also described an iterative process of rubric engineering to improve reliability of AI scoring and identified the patterns of scoring errors.

## Approach

### Setting

The study was conducted at two institutions: the Donald and Barbara Zucker School of Medicine at Hofstra/Northwell (ZSOM) and University of California at San Francisco School of Medicine (UCSF). Both schools administer end-of-course exams comprised of 100% OEQs in the pre-clerkship curriculum. ZSOM has been using OEQ exams for over ten years and UCSF for over seven years. The study was deemed exempt from review by each schools’ Institutional Review Board (Hofstra University IRB #20240307-SOM-OLV-1 and UCSF IRB #24–41146).

### Data

We identified 2 open-ended exam questions per institution that had previously been administered in the pre-clerkship curriculum to test medical knowledge and the integration of basic and clinical science. We chose questions representing diverse subjects (i.e., molecular biology, physiology, pathophysiology etc.) to support the generalizability of the findings. In addition, we chose questions with a range of student scores to assure that GPT-4 could score responses at all levels of the scoring rubric. The exams were administered at ZSOM in December 2023 to first-year medical students (MS1s; *N* = 99) and second-year medical students (MS2s; *N* = 101) and at UCSF in October 2023 to MS1 students (question 1H: *N* = 162; question 2H: *N* = 161). Faculty content experts scored the questions at their own institutions as per standard practice following exam administration, with one faculty member grading all responses to a single question.

### Scoring Rubrics

Assessment literature describes two rubric categories used for scoring OEQs: analytic and holistic rubrics [28]. ZSOM uses an analytic rubric for scoring, which comprises a list of specific components needed for each response to earn full or partial credit. Each component is worth one point with the total number of points summed for each question. The two questions from ZSOM were scored on a 4-point rubric. Holistic rubrics are typically used to make a single overall judgement about a student’s response based on multiple criteria. UCSF uses a holistic rubric consisting of a scale with three categories: meets expectations (5–6 points), borderline performance (3–4 points), and does not meet expectations (1–2 points). Each category includes specific anchors to guide graders on how to determine the grade based on information included in the answer. All vignettes, questions, rubrics and prompts are provided in Supplemental Appendix 1 (ZSOM) and Supplemental Appendix 2 (UCSF).

### Procedure

**Fig. 1:**
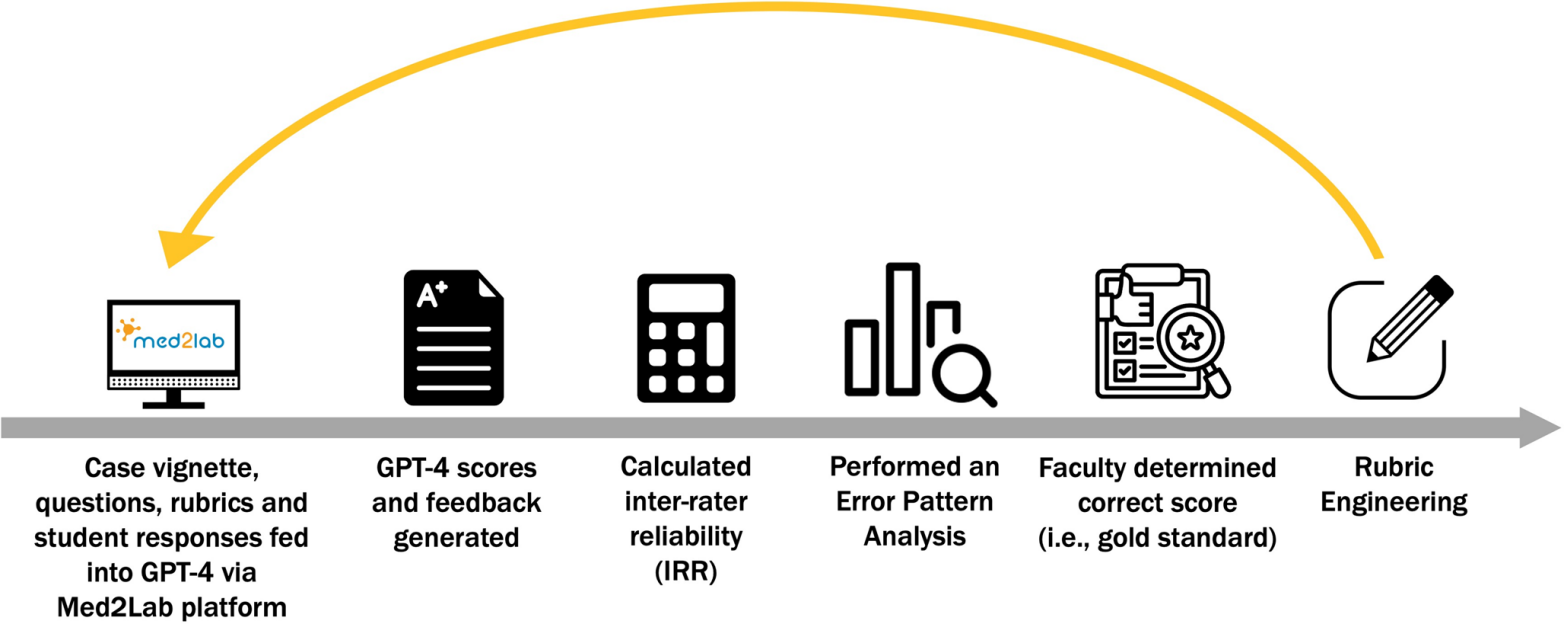
Detailed graphic of the steps in each iteration. A total of 3 iterations were performed

Between April 2024 and February 2025, three iterations of generative AI scoring were conducted. Each iteration included a series of tasks (Fig. 1). First, the case vignette, questions, rubric, and student responses for each question were entered into GPT-4 through the Med2Lab platform (https://cognitusai.com/) with the following instructional prompt: “YOU = a medical educator grading the QUESTION below, based on the RUBRIC. USER = student answering the QUESTION. You give a score to each answer, and an explanation for why you give such score.” For each student response, GPT-4 generated a score and an explanation consisting of 3–4 sentences of feedback. The scores were used to calculate the inter-rater reliability (IRR) between the faculty (i.e., human content expert) score and the GPT-4 score. The faculty then conducted an error pattern analysis [29], having at least two faculty members review student responses that were scored differently by faculty and GPT-4 scores, using the GPT-4-generated feedback to glean potential reasons for the discrepancy. During this process, the faculty were tasked with establishing a gold standard score by deciding which score was correct: the faculty score, GPT-4 score, or neither. If the faculty score was deemed incorrect, it was changed in subsequent iterations to reflect the corrected, gold standard score. The faculty then engineered the rubric by introducing deliberate targeted refinements based on the common scoring errors identified in the error pattern analysis. The adjusted rubric was used in subsequent iterations along with the original case vignette, questions, and student responses.

To adhere to privacy requirements and ethical considerations, the collected student responses were deidentified before inclusion into the Med2Lab system. Before uploading, all students’ written answers sent to the large language model were manually checked and verified to ensure that they did not contain any information that might reveal the identity of individual students. The answer texts were stored in a secure database hosted on Amazon Web Services (AWS). The database, as well as the deployment environment used to run the analysis, were password protected and accessible only to designated personnel. The model we used was OpenAI’s GPT-4o-2024-05-13. For ZSOM, the model was accessed via OpenAI’s cloud-based platform through the Med2Lab interface. For UCSF, we utilized a localized deployment of the same model hosted within UCSF’s secure, HIPAA-compliant private network.

### Statistical Analysis

Quantitative analysis of IRR was performed using IBM SPSS Statistics (SPSS Inc., Chicago, Illinois, USA, Version 28). The percent agreement and score distributions for each iteration are presented as the number (and percent) of scores. IRR between the faculty and GPT-4 scores at each site was calculated using Cohen’s weighted kappa (k_w_) with quadratic weighting. Cohen’s weighted kappa calculates the degree to which two raters agree on a scale from 0 (no agreement) to 1 (complete agreement) taking into account the degree of disagreement; larger differences between the raters will result in poorer agreement. Interpretation of the kappa index is as follows: < 0.20 = slight agreement, 0.20–0.40 = fair agreement, 0.40–0.60 = moderate agreement, 0.60–0.80 = substantial agreement, and > 0.80 = almost perfect agreement [30]. In addition, the most common discrepancies identified through the error pattern analysis were categorized and described.

## Results

We report on the agreement between faculty and GPT-4 scores and the error pattern analysis for the questions scored with the analytic rubric first, followed by the same analysis for questions scored with the holistic rubric. Examples of student responses and GPT-4 feedback for all four questions are presented in Table 1. All weighted kappa, percent agreement and additional IRR metrics for all four questions and three iterations are provided in Supplemental Appendix 3.

**Table 1:**
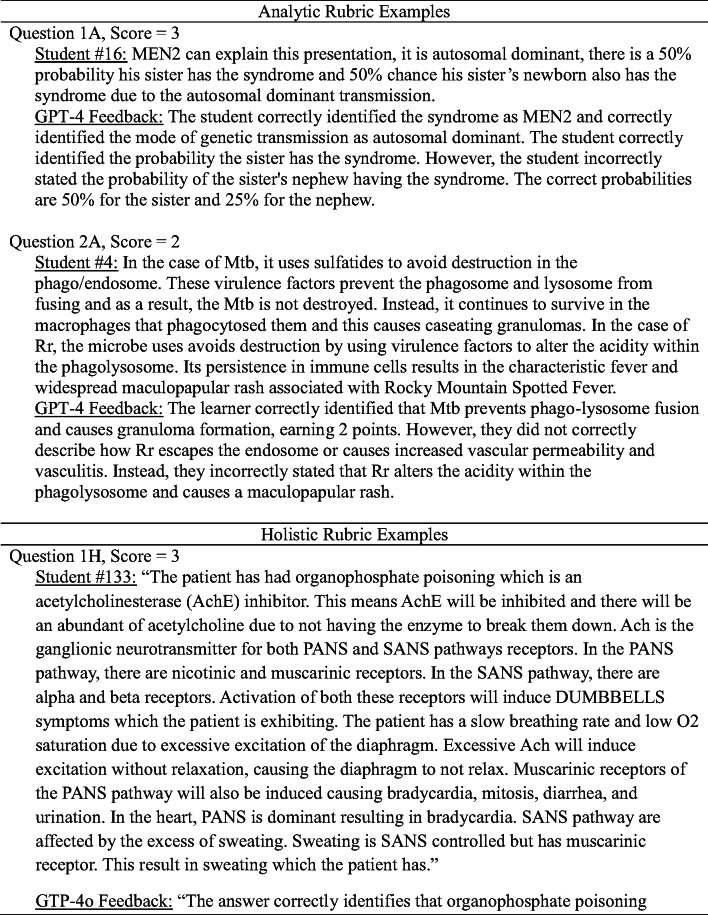

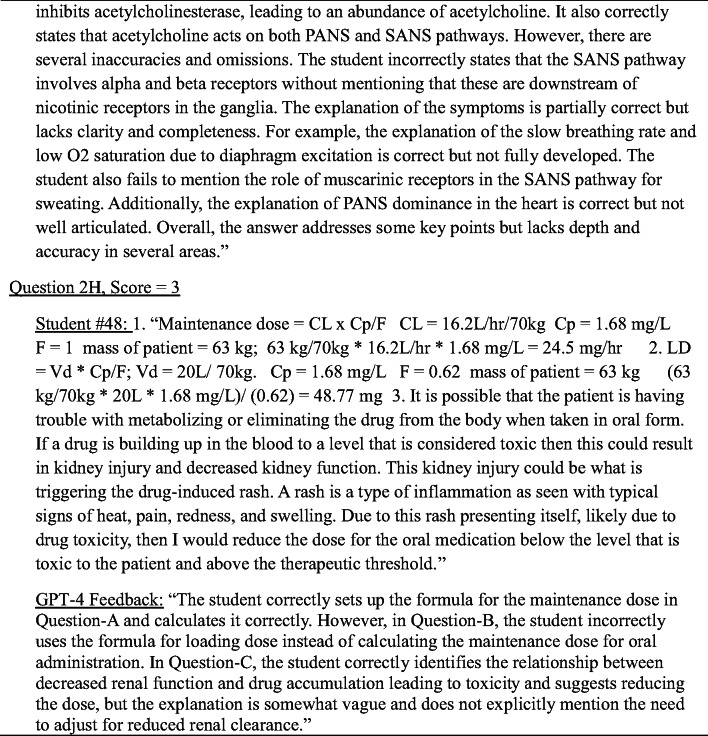
Most common reasons for discrepancies in scoring using the analytic rubric and representative examples (in italics)

### Analytic Rubric Results

#### Agreement between Human and AI

**Fig. 2:**
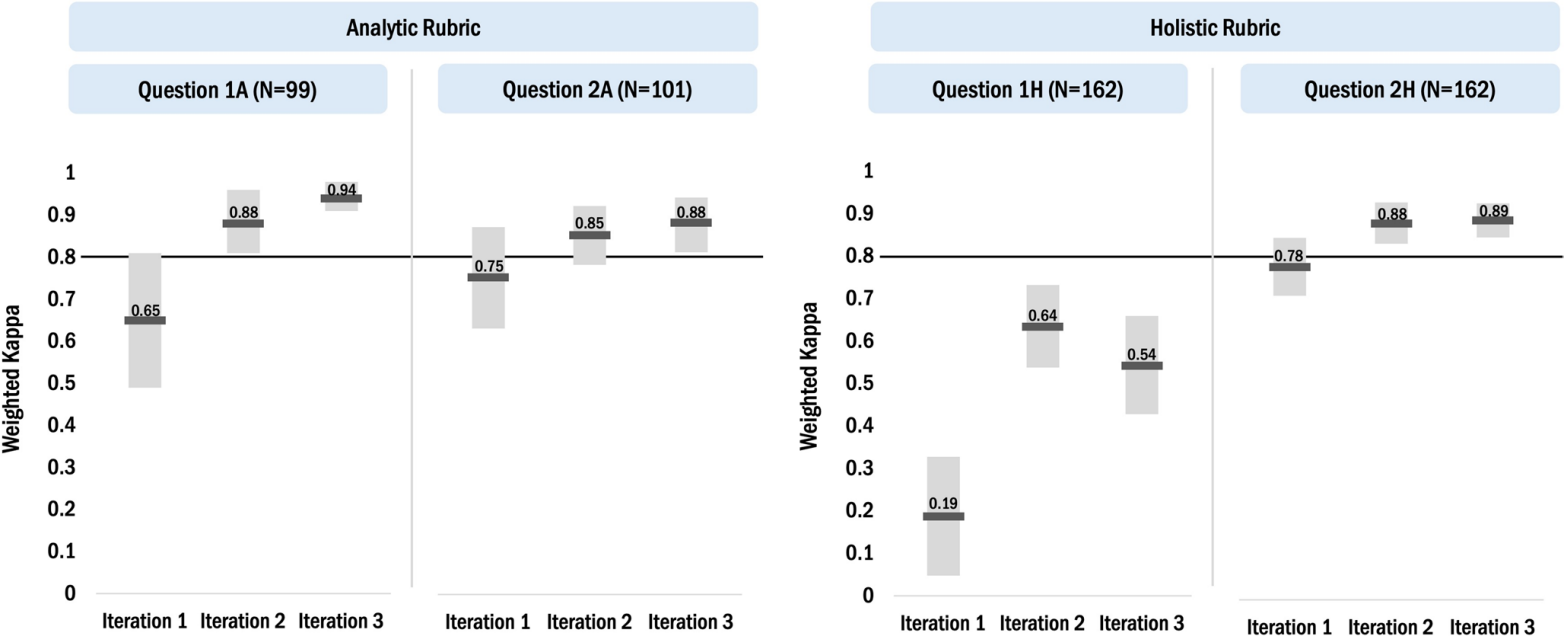
Weighted kappa (black bar) and 95% confidence interval (grey box) depicts inter-rater reliability (IRR) between the content expert and GPT-4 for each of three iterations. IRR for the analytic rubric is presented on the left and the holistic rubric on the right

The IRR between the human content expert scores and GPT-4’s scores is illustrated in Fig. 2. In the first iteration, IRR was substantial for questions 1A (k_w_=0.65, 95% CI: 0.49–0.81, 55% agreement) and 2A (k_w_=0.75, 95% CI: 0.63–0.87, 65% agreement). After refining scoring rubrics, the IRR increased to almost perfect agreement for both questions on iteration 2 (question 1A: k_w_=0.88, 95% CI: 0.81–0.96, 83% agreement; question 2A: k_w_=0.85, 95% CI: 0.78–0.92, 68% agreement) and iteration 3 (question 1A: k_w_=0.94, 95% CI: 0.91–0.98, 86% agreement; question 2A: k_w_=0.88, 95% CI: 0.81–0.94, 74% agreement).

**Fig. 3:**
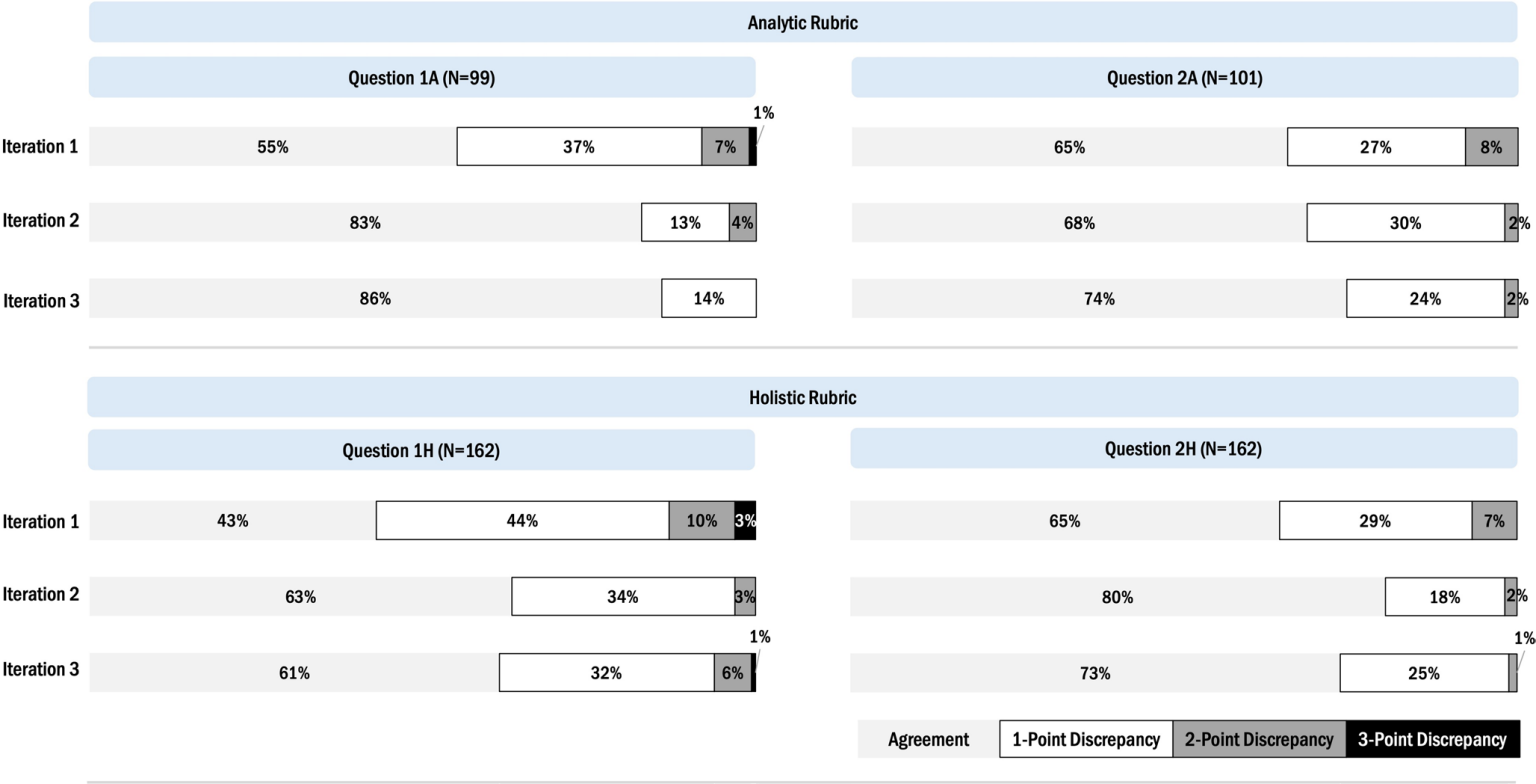
The cumulative percent of agreement and discrepancies by size identified during each of three iterations comparing faculty and GPT-4. Discrepancies for the analytic rubric questions are presented on the top and the holistic rubric questions on the bottom

To establish the degree of discrepancy between faculty and GPT-4 scoring, we quantified the difference in point value between the two graders (Fig. 3). Most were only one-point differences and there were smaller discrepancies after each iteration. After the 3rd iteration, all discrepancies for question 1A were only 1 point and for question 2A only two student responses had a 2-point discrepancy. Paired score data matrices and the distribution of scores assigned by faculty and GPT-4 are provided in Supplemental Appendices 4 and 5, respectively. Discrepancies occurred most often when faculty gave a score of 3 and GPT-4 gave a score of 2. For question 2A, discrepancies were also found in both directions when one grader gave a score of 3 and the other gave a score of 4. The matrices also visualize the evolution of faculty scores when they agreed with GPT-4. For example, for question 1A, GPT-4 gave a score of zero to four students, whereas faculty only gave a score of zero twice. However, after reviewing these discrepancies, faculty agreed that GPT-4 had correctly scored those responses and changed their scores to zero (as seen in iterations 2 and 3).

#### Question 1A Discrepancies

**Fig. 4:**
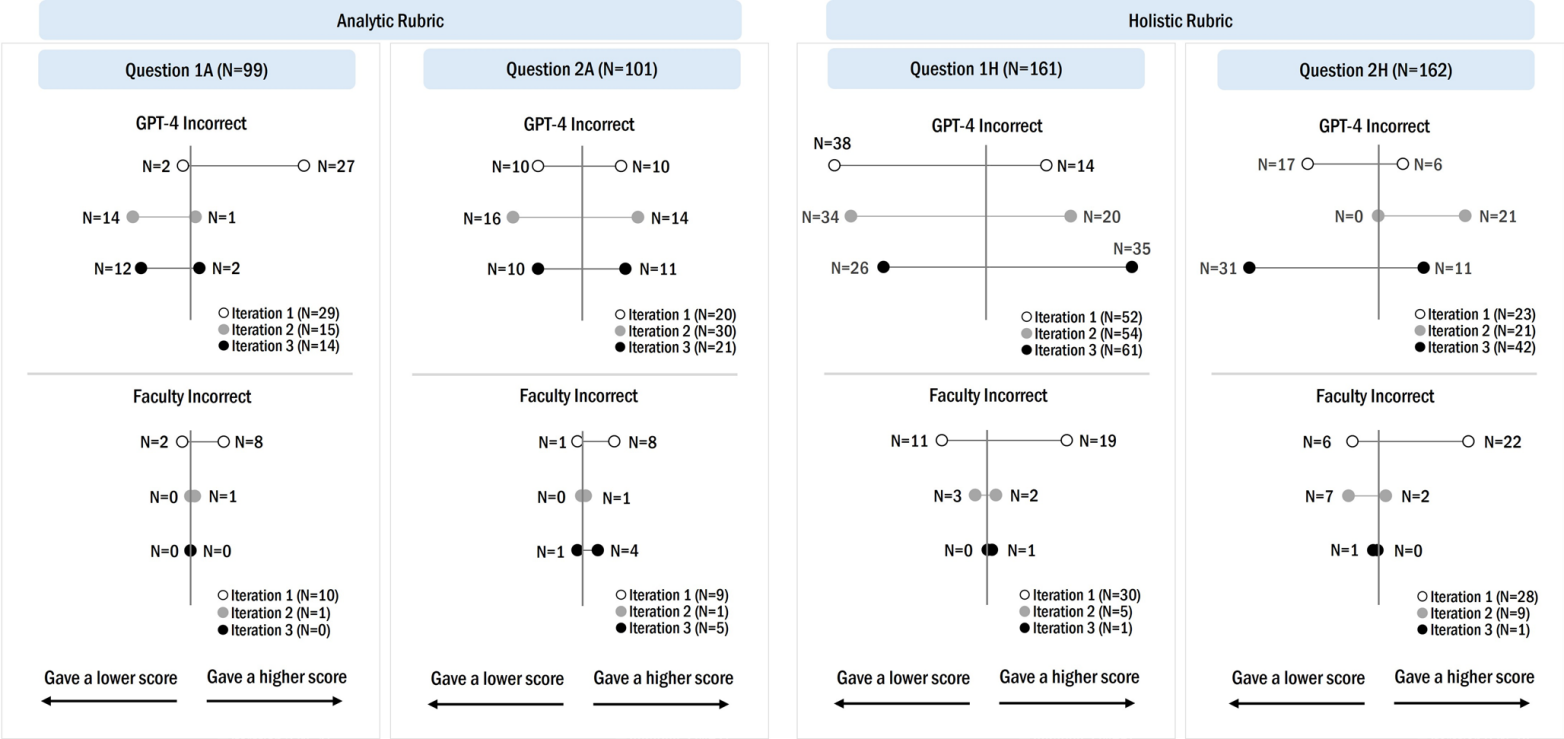
Figure showing how many discrepancies were due to errors in scoring by GPT-4 or faculty, as well as which direction the discrepancies occurred. Discrepancies for the analytic rubric are presented on the left and the holistic rubric on the right

Determinations of who was incorrect (i.e. GPT-4 or the faculty) and who assigned a higher vs. lower score are shown in Fig. 4. Question 1A presented a case of a male patient (Stephen) who presents to his physician with swelling in his neck and reporting a family history of neoplasms. The question asks students to identify the syndrome and genetic transmission, as well as the probability of his sister and his sister’s son (Alex) having the syndrome. The most common reasons for discrepancies are presented in Table 2. GPT-4 was unable to score accurately when students provided multiple possibilities. Also, GPT-4 often didn’t recognize when a student provided the incorrect transmission probability for Stephen’s sister or Alex.

**Table 2:**
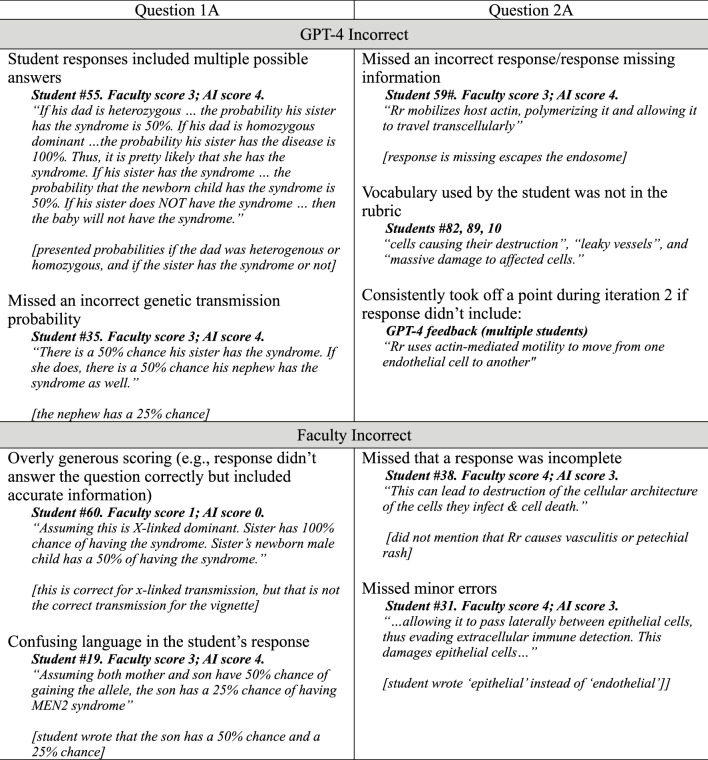
Most common reasons for discrepancies in scoring using the analytic rubric and representative examples (in italics)

We found that faculty were overly generous graders and awarded credit for responses that were true but did not answer the question. Students sometimes used confusing language, which also made it difficult for faculty to score.

There were a few occasions when both faculty and GPT-4 scored incorrectly (iteration 1: *N* = 5, iteration 2: *N* = 1, iteration 3: *N* = 0) and usually the errors reflected different types of oversights. For example, faculty didn’t give credit because the student did not quantify the sister’s probability (only writing that “*it is likely that his sister has the mutation*”) but failed to notice that the student also gave the incorrect probability for her child. GPT-4, in contrast, overlooked both issues and assigned the student a score of 4.

The evolution of the scoring rubric for question 1A is shown in Supplemental Appendix 1. When the student presented multiple scenarios specific to the sister’s genotype in determining the probability of the child, GPT-4 was able to correctly grade this by iteration 3. In the rubric for iteration 3, we added an instruction to give students one point only if one of the multiple possibilities present were correct, which reduced the occurrence of this type of discrepancy. All cases where GPT-4 missed the incorrect probability for the sister, the child, or both were resolved. In the rubric we added “Stephen’s sister” and “Alex, the son of the sister” to clarify how students may refer to the sister and the nephew, respectively, which appears to have addressed this issue. While cases with confusing language in iteration 1 were typically resolved, new discrepancies in subsequent iterations were flagged for confusing language.

#### Question 2A Discrepancies

Question 2A identified two pathogens (*Mycobacterium tuberculosis* [Mtb] and *Rickettsia rickettsii* [Rr]) and asked students to compare their mechanisms for avoiding destruction and to relate their intracellular growth to disease manifestations. The most common reasons for discrepancies are presented in Table 1. GPT-4 overestimated scores when it didn’t recognize that a response was missing a correct answer or had a wrong answer, with the most common error related to the Rr mechanism of avoiding destruction. GPT-4 also underestimated scores when vocabulary used by the student was not in the rubric but was accurate.

Faculty most often scored question 2A incorrectly if a student only provided one of the two critical answers (endothelial damage or vasculitis/rash), mistakenly giving full credit. Faculty also missed minor errors.

When both faculty and GPT-4 errors occurred (iteration 1: *N* = 6, iteration 2: *N* = 1, iteration 3: *N* = 0), scorers missed incorrect information but not in a consistent way. In one example, a student swapped the mechanism by which Mtb and Rr avoid destruction but faculty didn’t recognize this and GPT-4 missed that the student gave the incorrect mechanism for Rr avoiding destruction.

The evolution of the scoring rubric for question 2A is shown in Supplemental Appendix 1. There was some fluctuation between iterations in the number of times that GPT-4 simply overlooked an incorrect or missing answer, but clearly this type of error was difficult to address because the source of the error was not always evident. For example, GPT-4 continued to report that a student was describing the correct mechanism of avoiding destruction even when they were providing an incorrect response or missing a response. Discrepancies due to vocabulary were reduced in iteration 2, likely due to additions to the rubric providing alternative phrases, however when this was scaled back in iteration 3, these types of errors returned. In iteration 2, a model answer and alternative language section were added to the rubric provided GPT-4. However, GPT-4 then took off 1-point if students did not explicitly state that “*RMSF uses actin-mediated motility to move from one endothelial cell to another*,” which was language used in the model answer but not associated with a point value. This issue was not present in iteration 3 because this language was removed from the rubric. During iteration 3 there were six new discrepancies identified, of which five were errors in faculty scoring for various incorrect or correct responses that were missed.

### Holistic Rubric Results

#### Agreement between Human and AI

Figure 2 illustrates the IRR between faculty and GPT-4 scoring. In iteration 1, IRR was slight for question 1H (k_w_=0.19, 95% CI: 0.05–0.33, 43% agreement) and substantial for question 2H (k_w_=0.78, 95% CI: 0.71–0.85, 65% agreement). After refining scoring rubrics and prompts, the IRR increased for question 1H to substantial in iteration 2 (k_w_=0.64, 95% CI: 0.54–0.73, 63% agreement) but decreased to moderate in iteration 3 (k_w_=0.54, 95% CI: 0.43–0.66, 61% agreement). Question 2H’s IRR increased to almost perfect agreement in iteration 2 (question 2: k_w_=0.88, 95% CI: 0.83–0.93, 80% agreement) and remained stable in iteration 3 (k_w_=0.89, 95% CI: 0.85–0.93, 73% agreement).

Figure 3 depicts the discrepancies between faculty and GPT-4 scoring. Most were only one-point differences. For question 1H, there were few 2-point differences and no 3-point differences in iteration 2, but these increased in iteration 3. Both 1- and 2-point differences decreased for question 2H in iteration 2; 2-point differences further decreased in iteration 3 but 1-point differences increased. Paired score data matrices and the distribution of scores assigned by faculty and GPT-4 are provided in Supplemental Figs. 4 and 6, respectively. Discrepancies occurred most often between scores of 4 and 5 or 5 and 6 points and faculty were generally more likely to score a response one point higher than GPT-4.

#### Question 1H Discrepancies

Question 1H asked students to justify the diagnosis of drug poisoning by connecting the molecular actions of the drug on autonomic nervous system receptors to the observed findings. The most common reasons for discrepancies are presented in Table 1. GPT-4 overestimated scores when it did not recognize that responses were missing important information specified in the rubric, such as specific receptor locations or types or a description of dominant tone. GPT-4 underestimated scores when it took off additional points for acceptable errors. Some of these errors had already been specified in the rubric as acceptable, particularly those regarding ANS ganglia nomenclature that did not interfere with indication of overall understanding. GPT-4 also underestimated scores when it was looking for specific language that was in the rubric but not in the student’s response. GPT-4 was never able to identify a response that should have been scored a 2 (*N* = 3) and was only somewhat accurate at scoring a 3 (Iteration 2: *N* = 4/6, Iteration 3: *N* = 3/6).

**Table 3:**
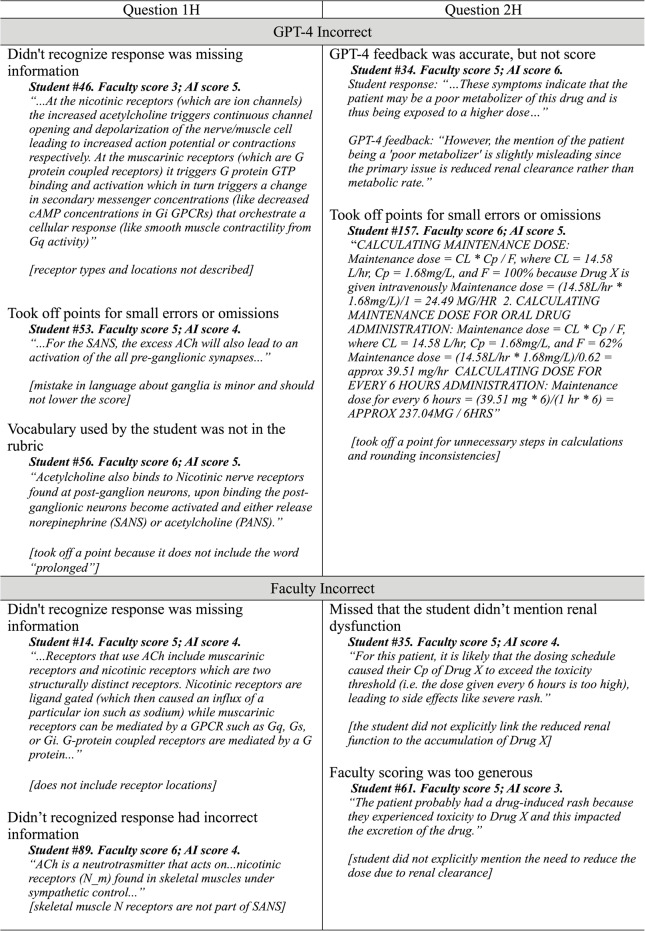
Most common reasons for discrepancies in scoring using the holistic rubric and representative examples (in italics)

When faculty scores were incorrect in iteration 1, the errors were often similar to GPT-4’s. Faculty overestimated scores when they did not recognize that student answers were missing important information or had incorrect information.

When both faculty and GPT-4 were incorrect (iteration 1: *N* = 10, iteration 2: *N* = 0, iteration 3: *N* = 0), the faculty missed an incorrect response at the same time GPT-4 was too strict or vice versa. On two occasions, both faculty and GPT-4 missed an incorrect response, giving higher scores than deserved.

The evolution of the scoring rubric for each iteration of question 1H is shown in Supplemental Appendix 2. Given the errors noted after iteration 1, it was apparent that more guidance was needed in the rubric to differentiate between a score of 5 and 6, providing examples that were acceptable to achieve a 6. Distinctions between 5 and 4 were also emphasized, with modifications to words in the rubric to prevent GPT-4 from assigning a 4 because of specific vocabulary (i.e. removed “prolonged” from “activation of Nn receptors” and “all” from “SANS and PANS ganglia” for a score of 4). Adding a model answer in iteration 3 proved to be counterproductive, resulting in a lower reliability. Many of the persistent discrepancies included feedback from GPT-4 about clarity of writing, which were difficult to address due to the vagueness of the feedback (e.g., *“minor issues with clarity and terminology.”*

#### Question 2H Discrepancies

Question 2H consisted of 3 sub parts: (A) calculating an IV maintenance dose (B) changing the IV to an oral dose every 6 h and (C) an application where students had to identify that reduced renal function led to reduced clearance requiring dose adjustment. The most common reasons for discrepancies are presented in Table 1. GPT-4 had difficulty assigning the correct score after properly identifying an incorrect response described in the feedback. GPT-4 was occasionally too strict, taking off one-point if extraneous information was included, or if the student used language to describe lowering the dose but did not explicated state lowering the dose.

The most frequent error faculty made was not taking enough points off for an incorrect answer to part C if students didn’t mention renal dysfunction, which warranted a 4. In contrast, faculty were too strict when students got multiple parts of the question wrong, including a calculation error (part B) and clinical reasoning error (part C). The calculation error was the same in most cases: students did not multiply the oral dose by 6 h. By contrast faculty were sometimes overly generous by allowing minor calculation errors.

In all seven cases where both faculty and GPT-4 scored incorrectly during iteration 1, faculty gave a score of 5 and GPT-4 gave a score of 3. In each case, the only error identified was the failure to explicitly point out renal disfunction, which meant that the highest score they could achieve was a 4. Errors by faculty and GPT-4 diminished over subsequent iterations (iteration 2: *N* = 2, iteration 3: *N* = 0).

The evolution of the scoring rubric for question 2H is shown in Supplemental Appendix 2. Language was added to the rubric to clearly define the criteria for each score. In addition, an important considerations section was included after iteration 1 and three model answers were included in iterations 2 and 3. After iteration 1, GPT-4 more accurately matched the feedback to the score, however GPT-4 struggled with this mismatch on low scores (i.e., 1–3). Discrepancies due to strict scoring were resolved by iteration 2, but these errors expanded in iteration 3. More than half of these discrepancies were originally mis-scored by faculty or both GPT and faculty in previous iterations and were concentrated on part C errors (e.g., giving a score of 3 when a student didn’t mention renal dysfunction). In iterations 2 and 3, GPT-4 missed minor incorrect responses, such as miscalculations in part A or not calculating the oral dose every 6 h for part B. Student responses that did not mention renal dysfunction that were incorrectly scored by faculty in iteration 1 were correctly scored by GPT-4 in iterations 2 or 3.

## Discussion

Our findings demonstrate that using an iterative process, GPT-4 can reliably score OEQs assessing medical knowledge. Three out of the four questions we examined had almost perfect inter-rater reliability after three iterations of edits to the scoring rubric. This is consistent with the literature on the reliability of generative AI [25, 26] and natural language processing models [21–24]. Our approach to incorporate multiple iterations of scoring and rubric engineering allowed us to improve reliability based on feedback from GPT-4. As a result, the final reliability measurements exceeded those reported in the literature when using a multi-point rubric [22, 24, 26]. This method also mimics real-world use of generative AI that requires prompt engineering to improve the quality of output [31].

We could not achieve almost perfect reliability with question 1H that was scored using a holistic rubric and was chosen specifically to be the most challenging question. Studies have traditionally found lower inter-rater reliability rates when using a holistic rubric [32–34]. Even though question 2H was also scored using a holistic rubric, the parts of the question that required dosage calculations could be scored analytically, perhaps contributing to a higher reliability than question 1H, which asked for a more synthetic answer. Reliability for question 1H improved considerably after the first iteration to the moderate reliability category. The improvement in reliability may have been limited by inclusion of conditional statements, such as “AND” or “OR,” which may have been challenging for GPT-4 to interpret. During iteration 3, we added a model answer to the prompt but this had a negative impact on GPT-4’s ability to score student responses. Morjaria and colleagues [26] also reported the lowest correlation between ChatGPT and faculty scores when using only a model answer or a combination of rubric and model answer. Providing faculty scores in the prompt can also introduce bias in generative AI’s scoring process [27].

Regardless of rubric type, most of the discrepancies were only a one-point difference, which is consistent with what has been reported in the literature [26]. Exact reliability may not be necessary when exams are graded in terms of pass/fail rather than a numeric score. Notably, 82% of US medical schools use a pass/fail grading system in the pre-clerkship curriculum [35]. Across all four questions GPT-4 sometimes failed to discriminate correct from an incorrect answer. In many cases it was unclear why this happened because GPT-4’s feedback did not necessarily align with the students’ response. GPT-4 was also unable to score properly when students included multiple possible answers. Scoring unnecessarily long responses that include extraneous information is challenging for human and non-human graders [25, 36]. It was also unable to score when it did not recognize vocabulary that was not in the rubric. When faculty scored incorrectly, it could have been due to a number of factors including fatigue [17], boredom [37] or subjective interpretation of the rubric [38]. There is also variability in how faculty approach scoring, which has been documented in the literature labeling scorers as hawks (strict graders) or doves (lenient graders) [39, 40].

GPT-4 generated detailed feedback that was useful for identifying errors in scoring. Ultimately, such feedback could provide students with timely [41] and personalized information about their performance on exam questions [42], which can drive future learning [43, 44]. Personalized student feedback can help facilitate the goal of precision medical education which would tailor curricular interventions based on each student’s needs [45, 46]. Though we did not rigorously assess the accuracy of GPT-4’s feedback in this study, faculty did note inaccuracies in feedback during the error pattern analysis which indicates that there is a risk of misleading students about their mastery of a topic [47, 48]. The quality of human feedback may exceed feedback from generative AI [49], therefore it is essential that AI feedback is continuously monitored by faculty and that students are equipped to evaluate its accuracy. Future studies will need to determine if the benefit of providing timely, individualized feedback outweighs occasional inaccuracies.

Prompt engineering has emerged as an essential skill needed to adjust the output generated from AI [31]. Our findings support the importance of rubric development and engineering to improve the reliability of OEQ scoring by generative AI. Another feature that parallels the technical literature on generative AI is the concept of having a “human in the loop” for quality assurance of the AI’s performance [50, 51]. In our study, there was human intervention checking for reliability and using feedback to determine patterns in scoring errors. When faculty reviewed the feedback generated by GPT-4 they were able to troubleshoot ways to improve the rubric for clarity and consistency. Identifying patterns in student responses can also provide faculty with feedback on topics they need to emphasize in future teaching.

This study provides a roadmap for medical educators to use generative AI to grade OEQs in practice. For example, prior exams can be used to evaluate the reliability of generative AI scores and to adjust scoring rubrics, as demonstrated in the current study. Although it requires a significant time investment up-front to establish reliability, generative AI can be used to score future exams with safeguards in place. It is essential that humans remain in the loop to check for validity (i.e., is AI providing accurate information), reliability (i.e., is AI scoring comparable to faculty scoring?), reproducibility (i.e., is AI providing consistent scores?), and bias (i.e., is AI providing biased information?) [52]. One strategy to confirm reliability is to have faculty score a subset of exams (e.g., low scoring or randomly selected exams) to ensure that they were accurately scored. Reproducibility can be assessed by having generative AI score the same exam multiple times. Validity and bias can be checked by reviewing generative AI’s personalized feedback for accuracy. If having generative AI score an entire exam is not feasible, generative AI may score OEQs that are more analytic and straightforward, reserving faculty scoring for more complex reasoning questions.

Our results underscore not only the potential of generative AI but also the importance of platform design; specifically, systems that integrate rubrics, prompts, and human adjudication in a reproducible workflow. Variability of generative AI’s scoring performance will depend on numerous factors, such as the OEQ, the rubric, and the generative AI model [53]. Furthermore, with the rapid introduction of new models it is unclear if reliability will remain robust on future generations of ChatGPT or other generative AI platforms, raising concerns about the generalizability of these findings. However, newer reasoning models may lead to improved reliability [54].

### Limitations

We acknowledge that there are several limitations to our study. Although we used questions scored using different types of rubrics and variable levels of difficulty, the analysis was limited to four questions at two US medical schools; therefore, it is unclear if the results will generalize to other assessment questions. We are confident that the iterative process we present will be transferable to other questions and institutions interested in using generative AI to score OEQs. The error pattern analysis was limited to the feedback provided by GPT-4, which was not always informative on why mistakes were made. We also did not know the cause of faculty errors. Thus, our analysis was more descriptive than analytical. Generative AI models lack transparency [55] and the underlying processes by which GPT-4 applied the rubric remain unknown beyond the prompt and rubric we provided.

### Conclusion

This project addressed the challenges of scoring OEQs for medical knowledge assessment. We found that GPT-4 could reliably score OEQs through an iterative process of rubric engineering. This process can be incorporated into assessment workflows which can ultimately reduce the time and effort dedicated by faculty to grading, freeing them to focus more on other priorities such as curriculum development and student engagement. Generative AI also has the potential to produce personalized feedback which can provide students with insight into the areas in which they can improve. Future research can focus on whether new iterations of generative AI models will maintain a high level of reliability and how to ensure the accuracy of generative AI’s feedback.

## Supplementary Information

Below is the link to the electronic supplementary material.

Supplementary File 1 Analytic rubrics & prompts for questions 1A and 2A. (DOCX 20.1 KB)

Supplementary File 2 Holistic rubrics & prompts for questions 1H and 2H. (DOCX 26.3 KB)

Supplementary File 3 Measures of inter-rater reliability (IRR) including weighted kappa (quadratic), weighted kappa (linear), Krippendorff’s Alpha, and Intraclass Coefficient (ICC), and percent agreement for all three iterations of GPT-4 scoring by question. Significant findings are in bold. (DOCX 17.2 KB)

Supplementary File 4 Data matrix showing faculty scores (columns) by GTP-4o scores (rows) for each run. Grey shading indicates agreement between faculty and GTP-4o scores. (JPG 1.02 MB)

Supplementary File 5 The distribution of scores for questions 1A and 2A for both faculty and GPT-4 for each iteration. (JPG 552 KB)

Supplementary File 6 The distribution of scores for questions 1H and 2H for both faculty and GPT-4 for each iteration. (JPG 543 KB)

## Data Availability

The datasets generated during and/or analyzed during the current study are available from the corresponding author on reasonable request.
