# Supplementary material for: Can Generative Artificial Intelligence Reliably Score Open-Ended Question Assessments in Undergraduate Medical Education?
Source: Med Sci Educ. 2026 Mar 3;36(3):1539–52. doi: 10.1007/s40670-026-02638-2 (PMC13355965; doi:10.1007/s40670-026-02638-2)
Supplement: Supplementary file 1 — Analytic rubrics & prompts for questions 1A and 2A. (DOCX 20.1 KB) [file 40670_2026_2638_MOESM1_ESM.docx]

ZSOM Question 1A

**System Prompt:** "YOU = a medical educator grading the QUESTION below, based on the RUBRIC. USER = student answering the QUESTION. You give a score to each answer, and an explanation for why you give such score."

**ITERATION #1 in black text. If any changes are made to the prompt or rubric, they are indicated in blue for ITERATION #2 or green for ITERATION #3.**

Vignette and Questions

Stephen is a 25-year-old man who comes to your office complaining of a swelling in his neck. While you are taking his family history, you discover that a number of family members on his father's side had a variety of neoplasms, almost all of them pheochromocytoma or medullary thyroid carcinoma. On further review of the family history, you find that these malignancies occur in every generation. Because you suspect a familial cancer syndrome, you draw a calcitonin level on Stephen, which is high, and confirm your suspected diagnosis through sequencing of the *c-RET* gene.

What syndrome can explain this presentation?

What is the mode of genetic transmission of this syndrome?

What is the probability his sister has the syndrome?

What is the probability that his sister’s newborn male child, Alex, also has the syndrome?

Rubric

Following are the correct answers for each of the above questions. Student should get 1 point for answering each question correctly (total score given is therefore 4 points). Do NOT give partial point for each concept (i.e. student should get either 0 or 1 point for each of the question):

- MEN2

- Phenotypically and genotypically autosomal dominant transmission

- There is 50% chance that sister has syndrome (learner has to specifically say 50% chance, or half, to get point for this, not any other estimate ("high", "above average", "very likely" etc.)

**ITERATION #2 & 3** - There is 50% chance that sister **(Stephen's sister)**has syndrome (learner has to specifically say 50% chance, or half, to get point for this, not any other estimate ("high", "above average", "very likely" etc.)

- There is 25% chance that the nephew will be affected ((learner has to specifically say 25%, or a quarter chance, to get point for this, not any other estimate ("high", "above average", "very likely" etc.)

**ITERATION #2 & 3**- There is 25% chance that the nephew **(Alex, the son of the sister)**will be affected ((learner has to specifically say 25%, or a quarter chance, to get point for this, not any other estimate ("high", "above average", "very likely" etc.)

**ADDED ON ITERATION #3:** For each of the latter 2 questions (about the probability of the sister and sister's newborn having the syndrome), if the learner offers an answer that constitutes multiple possibilities, the student gets full points if one of the possibilities is the correct one. (In this case, the father is very likely heterozygous because this is a rare disease - this underlying assumption can help the learner arrive at the right answer)

*ZSOM Question 2A*

**System Prompt:** "YOU = a medical educator grading the QUESTION below, based on the RUBRIC. USER = student answering the QUESTION. You give a score to each answer, and an explanation for why you give such score."

**ITERATION #1 in black text. If any changes are made to the prompt or rubric, they are indicated in blue for ITERATION #2 or green for ITERATION #3.**

Vignette and Questions

*Mycobacterium tuberculosis* (Mtb) and *Rickettsia rickettsii* (Rr) are both intracellular pathogens but they cause very different diseases.

Compare and contrast the mechanism by which Mtb and Rr avoid destruction within the phago/endosome of host cells. For each microbe, relate the nature of their intracellular growth to a pathological feature of the disease they cause.

Rubric

Give points if the learner mentioned each of the following concepts (maximum score is 4 points). Do NOT give partial point for each concept (i.e. student should get either 0 or 1 point for each of the 4 concepts):

- Mtb prevents phago-lysosome fusion (1 point)

- Rr escapes the endosome (1 point)

**ITERATION #3** - Rr escapes the endosome (1 point) (the students have to explicitly mention that Rr escapes or gets out of the endosome or phagosome to get full points for this question - i.e. please do not give point for this if the student simply describes a mechanism, such as movement between cells, without explicitly mention of the fact that Rr escapes the endosome)

- Mtb causes granuloma formation (1 point - student must mentioned "granuloma formation" to get 1 point for this concept)

**ITERATION #2** - Mtb causes granuloma formation (1 point - student must correctly mentioned or described the concept of "granuloma formation" to get 1 point for this concept)

**ITERATION #3** - Mtb causes granuloma formation (1 point - student must correctly mentioned "granuloma formation" to get 1 point for this concept)

- Rr causes increased vascular permeability (or "endothelial damage") AND vasculitis (or "petechial/nonblanching rash") (1 point).

**ITERATION #2 & 3** - Rr causes increased vascular permeability ("endothelial damage" is an acceptable alternative description of this mechanism) AND vasculitis ("petechial/nonblanching rash" is an acceptable alternative for vasculitis) (1 point). The students must described both the correct mechanism AND the correct resulting presentation to get the full point.

**ADDED ON ITERATION #2** - IMPORTANT CONSIDERATIONS: In answer to this question, it's ok for the students to use different ways to talk about the following concepts:

- Endosome may also be: Phagosome

- Actin-mediated motility may also be:

--Actin polymerization and movement/motility

--Actin-based polymerization

--Actin filaments/fibers

--Actin rockets

--Move from cell to cell via/through actin filaments

- Motility/movement may also be:

--Shoot from cell to cell

-Endothelial damage/destruction may also be:

--Tight junction damage

--Leaky vessels

**ADDED ON ITERATION #3** - IMPORTANT CONSIDERATIONS:

In answer for this question, it's ok for the students to use different ways to talk about the following concepts:

- Endosome may also be: Phagosome

- Endothelial damage/destruction may also be:

--Tight junction damage

--Leaky vessels
