# Supplementary material for: Can Generative Artificial Intelligence Reliably Score Open-Ended Question Assessments in Undergraduate Medical Education?
Source: Med Sci Educ. 2026 Mar 3;36(3):1539–52. doi: 10.1007/s40670-026-02638-2 (PMC13355965; doi:10.1007/s40670-026-02638-2)
Supplement: Supplementary file 2 — Holistic rubrics & prompts for questions 1H and 2H. (DOCX 26.3 KB) [file 40670_2026_2638_MOESM2_ESM.docx]

UCSF Question 1H

**System Prompt:** "YOU = a medical educator grading the QUESTION below, based on the RUBRIC. USER = student answering the QUESTION. You give a score to each answer, and an explanation for why you give such score."

**ITERATION #1 in black text. If any changes are made to the prompt or rubric, they are indicated in blue for ITERATION #2 or green for ITERATION #3.**

Vignette and Questions

A 31-year-old man (he/him) who is a farm worker was exposed to chlorpyrifos, an organophosphate (OP) insecticide used in California on a wide range of crops.

The exposure occurred approximately 10 minutes ago. The hospital was notified, and the emergency medical technicians (EMTs) were able to arrive quickly on the scene in full personal protective equipment (mask, gown, gloves, etc.). The patient is known to be in good health and is not taking any medications.

He is awake, but is confused and appears to be in distress. Physical exam reveals that he is sweating profusely, his eyes are watering, his pupils are small, and he is salivating. He has difficulty breathing with copious secretions (resulting in crackles on examination) and weak, shallow breaths. He has lost control of his bowels and bladder. Vital signs are as follows:

Temperature: 98 F (normal 97.8-99)

HR: 45 beats per minute (normal 60-100)

BP: 95/85 mm/Hg (normal <120/80)

O_2_ sat: 90% (normal = 95-100)

RR: 8 (normal 12- 18 breaths per minute)

Justify a diagnosis of organophosphate poisoning by connecting the molecular actions of organophosphates on key ANS receptors to the observed findings. Your answer should include: 1) the mechanism of action of an organophosphate (OP), 2) how the OP affects ANS signaling at two structurally distinct receptor classes expressed in different locations and 3) how the OP affects both arms of the autonomic nervous system (include two of this patient’s symptoms and explanations for why those symptoms demonstrate which arm dominates in that tissue).   (Length guideline: 2 to 3 paragraphs)

Rubric

Give a score between 2 (worst) and 6 (best), based strictly on the following criteria:

* Score = 6: Addresses each part of the question, including MOA, roles of N/ion channel receptors and M/GPCRs and correct locations, choosing 2 findings, explaining conceptually why they occur, & includes SANS & PANS arms of ANS and notes PANS is dominant.

**ITERATION #2** * Score = 6: Addresses each part of the question, including MOA, roles of Nn and M receptors and correct locations, choosing 2 findings, explaining conceptually why they occur, & for any symptom in a tissue that has both SANS & PANS innervation notes that PANS is dominant. (Students do not necessarily need to say that PANS is dominant in “most tissues” to get a 6, only to be clear about dominance in the tissue they discuss. A description of SANS as “dominant” in mediating sweating or vasoconstriction is acceptable for a 6 even though thermoregulatory sweat glands and blood vessels are not innervated by PANS ganglia. Students also do not need to name that Nn receptors are ion channels or that M receptors are GPCRs, though if they do describe these incorrectly this should be described as an error in the feedback and may warrant a 5 score. Students do not need to name the G proteins associated with particular M receptors and if they do we should not take a point off even if it is inaccurate, though this can be described as a minor error in the feedback. If students have described G protein-mediated signaling in detail, there need not be any comment about it and it should not detract from their score.)

**ADDED ON ITERATION #3:** N receptors do not need to be named as “Nn” as long as the answer clearly indicates the location of the receptors is on neurons or nerves (or postsynaptic cells or postganglionic cells). The answer does not need to explicitly say that N receptors are located all “all ANS ganglia” as long as they have indicated somewhere in their answer that N receptors are present on neurons (or postsynaptic cells or postganglia or postganglionic neurons) of the parasympathetic (PANS) and sympathetic nervous system (SANS)

Note that if the student describes activation of N receptors at SANS ganglia and activation of N receptors at PANS ganglia in very different parts of the answer this shouldn’t bump them to a 5 from a 6.   

* Score = 5: Above (criteria score = 6) but with small errors; including saying OPs bind with “high affinity” OR description of dominance of PANS is slightly off or less than completely formalized: Errors in ANS phys/anatomy terminology (common – talking about N receptors at “preganglionic neuron” when mean “postganglion”) OR includes reference to M receptors in overview but doesn’t connect directly to role in responses OR gets one receptor description incorrect (calls alpha/beta receptors ion channels for example).

**ADDED ON ITERATION #2 & 3**: All of these should be described as minor errors in the feedback. Note that if the student describes activation of Nn receptors at SANS ganglia and activation of Nn receptors at PANS ganglia in very different parts of the answer this shouldn’t bump them to a 4 from a 5.

* Score = 4: Addresses main parts of the question, but does not directly or accurately address dominance OR does not explicitly address prolonged activation of Nn receptors on all ganglia OR has errors or omissions about M receptors (e.g. Ach acts directly only at N receptors and not M)

**ITERATION #2:** Score = 4: Addresses main parts of the question, but does not directly or accurately address dominance OR does not explicitly address activation of Nn receptors on both SANS and PANS ganglia OR has errors or omissions about M receptors (e.g. Ach acts directly only at N receptors and not M) OR suggests that the somatic nervous system and Nm activation is under SANS control OR has combinations of the minor errors described in the “5” score.

**ADDED ON ITERATION #3:** For example, student omits a reference to or description of a tissue that has dual innervation by PANS and SANS, and omits a description of M receptors on tissues that are targeted by ANS innervation.

* Score = 3: Similar to criteria of score = 4, but if both are missing OR gets MOA wrong but accurately explains rest (based on incorrect MOA) OR more significant errors related to terminology/concepts re: activation of Nn receptors at all ganglia OR one part of the question not answered at all

**ITERATION #2: unchanged**

**ADDED ON ITERATION #3**: (for example omission of MOA)

* Score = 2: major, fundamental errors in ANS physiology, even if MOA and dominance concepts are there; additional combinations of errors described in 3

**ITERATION #2:** unchanged

**ADDED ON ITERATION #3:** Model Answer:

1) Organophosphates (OPs) are acetylcholinesterase inhibitors - indirect-acting cholinomimetics that inhibit acetylcholinesterase throughout the body. This increases the half-life of Ach in all synapses, leading to prolonged signaling at all nicotinic (N) and muscarinic (M) receptors, in both arms of the ANS (parasympathetic and sympathetic) and at skeletal muscle.

2) Receptors affected are nicotinic receptors (Nn at postsynaptic neuronal cell bodies in all ANS ganglia, Nm at skeletal muscle) and muscarinic receptors (at all ANS target tissues).

3) Increased Ach results in both the SANS and PANS being activated, along with skeletal muscle in somatic NS, since Ach acts at nicotinic (Nn) receptors in PANS and SANS ganglia. The end result of this activation depends on the target tissue of the postganglionic neuron and the dominant arm in that tissue. Most of the symptoms are PANS-dominant (“rest and digest”) effects in tissues that are dually innervated, reflecting activation of M receptors in those tissues - which explains the diarrhea, urination, miosis, lung (secretions and difficulty breathing related to bronchoconstriction), bradycardia, tears, and salivation. Sweating is SANS mediated, also via M receptors on the target tissue.  (The patient also exhibits decreased oxygen sat and decreased respiratory rate which could be attributed to overstimulation of skeletal muscle; confusion could be attributable to CNS excitation). Collectively the symptoms that indicate organophosphate poisoning are “DUMBBELSS”: diarrhea, urination, miosis, bronchoconstriction, bradycardia, excitation, lacrimation, salivation, sweating. It was important to connect the molecular effect of OP on ANS signaling directly to two of these. Given the need to discuss dominance, at least one effect in a tissue dually innervated by PANS and SANS should be explained.

Incidentally, nicotinic receptors are ion channels that transmit a signal by allowing ions to cross the plasma membrane, altering the local voltage. Muscarinic receptors are GPCRs that transmit signals by coupling to G proteins and producing intracellular second messengers. However this information did not need to be included for a ‘meets’ score.

CONTEXT:

Scores 5 & 6 constitute "Meets Expectation"

Scores 3 & 4 constitute "Borderline achievement of expectations"

Scores 0, 1 & 2 constitute "Does not meet expectations"

An omission that was a critical distinction between ‘meets’ and ‘borderline’ was the description of HOW both arms of the ANS are activated by prolonged Ach signaling at Nn receptors, which required that the locations of N receptors in all (both SANS and PANS) ganglia be included (“the synapse” was not precise enough), which is the reason for the question about dominant tone. Another common issue was lack of a complete discussion about PANS dominance in the tissue exhibiting the symptom(s) chosen (by for example omitting a symptom in a tissue that is innervated by both PANS and SANS and describing this concept), or inaccurately attributing PANS dominance to the involvement of both Nn and M receptors in PANS signal transmission. Omitting locations of receptors (again, Nn at all ganglia, with M downstream at target tissues of PANS innervated tissues) was another reason for a ‘borderline’ rather than a ‘meets.’

UCSF Question 2H

**System Prompt:** " SCORING INSTRUCTIONS: Score the answer for this question using the RUBRIC below. The following MODEL ANSWERS provides an ideal example and should all get 6 points."

**ITERATION #1 in black text. If any changes are made to the prompt or rubric, they are indicated in blue for ITERATION #2 or green for ITERATION #3.**

Vignette and Questions

A 40-year-old nonbinary person (he/they) with no significant past medical history now comes to your clinic reporting high fevers (up to 103 degrees F; normal range 97.8-99), painful cough, and severe fatigue. These symptoms developed rapidly over the past three days. On physical exam today, the patient’s blood pressure is 121/81 mm Hg (normal <120/80), heart rate 105 beats/min (normal 60-100), respiratory rate 22 breaths/min (normal 12-20) and temperature 101 degrees F. During lung examination, he says it hurts to breathe in deeply. They are a teacher and all their PPD skin tests for work clearance have been negative. There is no personal history of tuberculosis (TB) or history of TB exposure.

The patient is admitted and will receive an intravenous (Drug X) as part of the regimen to manage their disease. The target concentration of Drug X for this patient is 1.68 mg/L.

Pharmacokinetic information about Drug X:

Vd = 20 L/70kg

Cl = 16.2 L/h/70kg (70 % renal, 30 % metabolic)

Oral availability (F) = 62%.

1) Calculate an IV maintenance dose to maintain the target plasma concentration of Drug X at 1.68 mg/L. The patient weighs 63 kg. Please show your calculations and the formulas you use. (Length guideline: 2 to 3 lines)

2) Upon discharge to home, you prescribe Drug X as an oral tablet once every 6 hours to maintain the same average plasma concentration as in the hospital (1.68 mg/L). The instructions to the patient say to take one tablet every 6 hours for ten days. How much Drug X must be in each tablet to maintain the average plasma concentration of 1.68 mg/L? You can assume that he indeed takes one tablet every 6 hours, as prescribed. (Length guideline: 2 to 3 lines).

3) Two days later (ii.e. before the ten days are over) they return to the ED with signs and symptoms of acute kidney injury, resulting in decreased renal function. The exam is also notable for a new severe rash. Rash is a known serious side effect of Drug X. Explain why the patient developed this side effect (i.e., drug-induced rash) now and how this might change your treatment plan. Assume it is essential that they remain on drug therapy with drug X until they completed a full ten days of therapy. (Length guideline: a few sentences, no calculations needed for C -just a general suggestion)

Rubric

Choose a score based on the rules below:

**ITERATION #2 & 3** - Give a score between 1 (worst) and 6 (best), based strictly on the following criteria:

- 6 points (Meets expectations): Use correct formulas and do calculations correctly.

**ADDED ON ITERATION #2**: AND suggests renal dose adjustment or other drug.

**ITERATION #3:** Use correct formulas and does calculations correctly AND links drug toxicity to reduced renal clearance suggests dose reduction or other drug. NOTE: Important mentioning reduced renal clearance drug accumulation and any form of dose reduction warrants a 6.

- 5 points (Meets expectations):if there are slight mistakes in calculations that do not involve orders of magnitude/units (i.e. plug in a slightly different number for one of the values, come up with an incorrect number within one of the calculations but set-up looks correct) OR a substitution of a unit in the answer but had it correct internally (L/hr or mg/L at the end of A) AND suggests renal dose adjustment or other drug linking renal failure to drug accumulation and drug toxicity OR A&B correct and noted accumulation due to reduced CL but small error in how to dose adjust.

**ITERATION #2 :** Score = 5: Above (criteria score = 6) but if there are slight mistakes in calculations that do not involve orders of magnitude/units (i.e. plug in a slightly different number for one of the values, come up with an incorrect number within one of the calculations but set-up looks correct) OR a substitution of a unit in the answer but had it correct internally (L/hr or mg/L at the end of A)

**ADDED ON ITERATION #3:** (i.e., If numbers do not match model answer the highest possible score is a 5)

- 4 points (Borderline achievement of expectations): Set up formulas correctly but leave out units OR have units incorrect for both OR Don’t adjust for weight in A OR Don’t account for reduced bioavailability in B OR Don’t calculate the dose per 6 hrs in B OR have mistakes in units that involve orders of magnitude

**ITERATION #2**: unchanged

**ADDED ON ITERATION #3:** and links drug toxicity to reduced renal clearance suggests dose reduction or other drug.

- 3 points (Borderline achievement of expectations): Just say stop/dose reduce drug X without explanation OR Written so that link between renal failure and rash might be implied without explaining that drug accumulates

**ADDED ON ITERATION #2** - Score = 3: Similar to criteria of score = 4, but…

**ITEREATION #3** - Score = 3: Similar to criteria of score = 4, but just say stop/dose reduce drug X without explanation AND written so that link between renal failure and rash /toxicity due to drug accumulation is not clear or missing

- 2 points (Does not meet expectations): Wrong formulas OR wrong numbers and no units OR No relation between renal failure and rash due to drug accumulation

**ITERATION #2:** unchanged

**ITERATION #3 -** Score = 2: Use wrong formulas OR wrong numbers AND no units for both 1 & 2 AND no relation between renal failure and rash due to drug accumulation

- 1 point (Does not meet expectations): Made no change to therapy OR Made wrong change to therapy

**ITERATION #2** - Score = 1: Similar to criteria of score = 2, but also suggests No change to therapy OR otherwise making wrong change to therapy

**ITERATION #3 -** Score = 1: Similar to criteria of score = 2, AND fails to suggest change to therapy OR otherwise making wrong change to therapy

Score = 1: NO response to at least 2 of the 3 sub parts of the question ( 1, 2 or 3)

**ADDED ON ITERATION #2 and #3:** IMPORTANT:

- The units are case insensitive, and can be abbreviated. (for example: MG or mg are both acceptable as milligram, MG/HOUR and mg/h are equivalent).

- It is acceptable for students to omit / miss some units in the calculation, as long as their final values in answering for Question-A and Question-B have the right units

- For Question-C - as long as the student indicates that they take into consideration decreased in renal clearance (or renal function), and that the dose should be decreased, that should be adequate for answering this question. There is NO need for student to explain in greater details, or to calculate a new adjusted dose.

**ADDED ON ITERATION #3:**
MODEL ANSWER #1:
1. IV maintenance dose should be: 24.5 mg/h. • MD = (Cp x CL) / F • MD = [(1.68 mg/L) x (16.2 L/h/70kg x 63kg)]/1.0 • MD = 24.5 mg/h
2. Each tablet must contain 237 mg.
Oral MD = IV MD/0.62 = 39.51 mg/h • Pill dose = MD x 6 h = 39.5 mg/h x 6 h = 237 mg
3. Most likely the reduced renal function allowed concentrations of drug X to build up causing toxicity (rash). Treatment options are 1) choose another drug if available or 2) reduce dose to maintain therapeutic levels using lower doses.

MODEL ANSWER #2:
1. I'll use maintenance dose formula, which is (CL x Cp)/F. CL is clearance, Cp is target concentration, F is the availability. First I calculate clearance for the patients weight, which comes out to 16.2 L/h x 63kg/70kg. This gives me a CL of 14.58L/h. Plug the numbers in to get [(14.58 L/h) x (1.68mg/L)]/1 = 24.5 mg/hr
2. The maintenance dose per hour taken orally is (24.5mg/hr)/.62 = 39.5 mg/hr. Taken every 6 hours we'd want each pill to be 237 mg.
3. I'd change the treatment plan to decrease the dosage. If there is kidney injury, we likely have lower clearance in this patient and there may have been some toxicity involved. Eosinophils may have responded to this drug toxicity and contributed to the rash and inflammation.

MODEL ANSWER #3:
1) Maintenance dose = CL*Cp/F=14.58L/h*1.68mg/L = 24.5mg/hr F=100% Cp=1.68mg/L CL=16.2L/h*(63kg/70kg) = 14.58L/h
2) 24.5mg/hr/0.62=39.5mg/hr for oral*6 hrs = 237mg Drug X in each tablet
3) This patient developed this side effect because his kidneys have diminished function. This means that less of the drug is eliminated via the kidneys/through renal excretion than initially expected (70% of drug is supposed to be eliminated through renal excretion). As a result, more of Drug X stays in the body than normal/safe, leading to drug toxicity, leading to the rash developing. Lowering the dose of Drug X to account for the new clearance rate of the drug would be necessary. This would result in the normal/safe amount of drug staying in the body, and hopefully would prevent drug toxicity.

IMPORTANT CONSIDERATIONS - The following are common Student Misconceptions that will affect the score:
1) using loading dose instead of maintenance dose
2) missing reduced clearance due to reduced renal function
3) Failing to propose to lower the dose in face of reduced clearance to achieve the level that is therapeutic and not toxic
4) some suggested more frequent dosing to avoid higher peaks but see 3) you also need the reduce the actual dose you give when CL is reduced.
