# Supplementary material for: Can Generative Artificial Intelligence Reliably Score Open-Ended Question Assessments in Undergraduate Medical Education?
Source: Med Sci Educ. 2026 Mar 3;36(3):1539–52. doi: 10.1007/s40670-026-02638-2 (PMC13355965; doi:10.1007/s40670-026-02638-2)
Supplement: Supplementary file 3 — Measures of inter-rater reliability (IRR) including weighted kappa (quadratic), weighted kappa (linear), Krippendorff’s Alpha, and Intraclass Coefficient (ICC), and percent agreement for all three iterations of GPT-4 scoring by question. Significant findings are in bold. (DOCX 17.2 KB) [file 40670_2026_2638_MOESM3_ESM.docx]

Supplemental 3. Measures of inter-rater reliability (IRR) including weighted kappa (quadratic weights), weighted kappa (linear weights), Krippendorff’s Alpha, and Intraclass Coefficient (ICC, two-way mixed effects model with absolute agreement), and percent agreement for all three iterations of GPT-4 scoring by question. Significant findings are in **bold**.

|  | Weighted Kappa  (Quadratic) | Weighted Kappa (Linear) | Krippendorff’s Alpha | ICC | Percent Agreement |
| --- | --- | --- | --- | --- | --- |
| Question 1A (Analytic Rubric) | | | | | |
| Iteration 1 | .65 (.49-.81) | .48 (.33-.62) | .50 (.34-.64) | .67 (.54-.77) | 55% |
| Iteration 2 | **.88 (.81-.96)** | **.82 (.74-.91)** | **.86 (.78-.94)** | **.89 (.85-.93)** | 83% |
| Iteration 3 | **.94 (.91-.98)** | **.88 (.82-.94)** | **.94 (.91-.97)** | **.95 (.92-.96)** | 86% |
| Question 2A (Analytic Rubric) | | | | | |
| Iteration 1 | .75 (.63-.87) | .63 (.50-.75) | .66 (.53-.78) | .75 (.65-.83) | 65% |
| Iteration 2 | **.85 (.78-.92)** | .72 (.62-.81) | .79 (.70-.86) | **.85 (.78-.89)** | 68% |
| Iteration 3 | **.88 (.81-.94)** | .77 (.68-.86) | **.85 (.77-.92)** | **.88 (.82-.91)** | 74% |
| Question 1H (Holistic Rubric) | | | | | |
| Iteration 1 | .19 (.05-.33) | .14 (.04-.24) | .18 (.01-.35) | .19 (.04-.34) | 43% |
| Iteration 2 | .64 (.54-.73) | .51 (.41-.62) | .63 (.53-.72) | .64 (.54-.72) | 63% |
| Iteration 3 | .54 (.43-.66) | .46 (.35-.57) | .57 (.46-.67) | .55 (.43-.65) | 61% |
| Question 2H (Holistic Rubric) | | | | | |
| Iteration 1 | .78 (.71-.85) | .63 (.56-.71) | **.82 (.76-.87)** | **.80 (.74-.85)** | 65% |
| Iteration 2 | **.88 (.83-.93)** | **.80 (.73-.86)** | **.87 (.81-.92)** | **.90 (.86-.92)** | 80% |
| Iteration 3 | **.89 (.85-.93)** | .77 (.71-.83) | **.89 (.84-.92)** | **.89 (.86-.92)** | 73% |
