# Supplementary figures and images for: Can Generative Artificial Intelligence Reliably Score Open-Ended Question Assessments in Undergraduate Medical Education?
Source: Med Sci Educ. 2026 Mar 3;36(3):1539–52. doi: 10.1007/s40670-026-02638-2 (PMC13355965; doi:10.1007/s40670-026-02638-2)

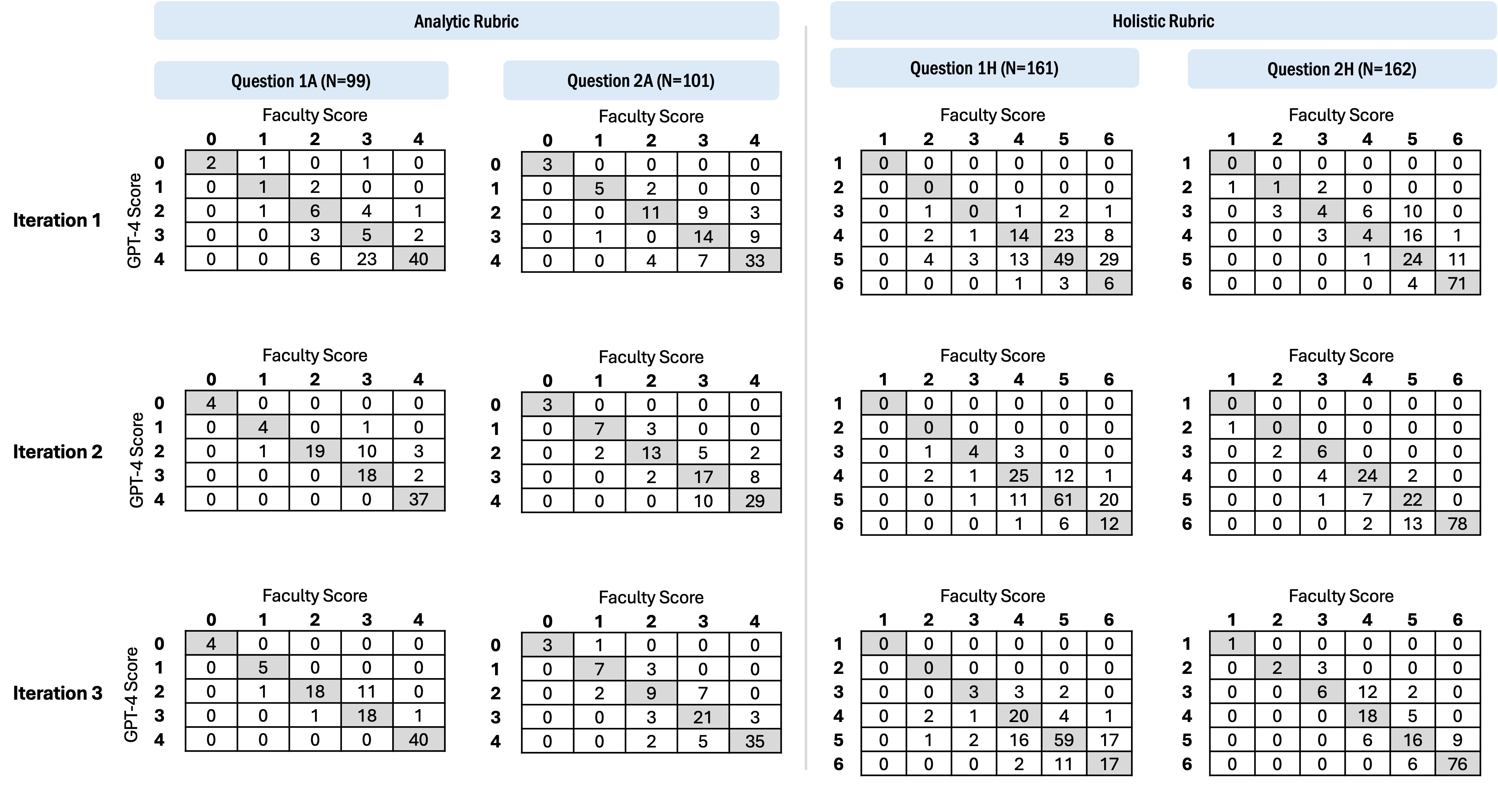

Supplement: Supplementary file 4 — Data matrix showing faculty scores (columns) by GTP-4o scores (rows) for each run. Grey shading indicates agreement between faculty and GTP-4o scores. (JPG 1.02 MB) [file 40670_2026_2638_MOESM4_ESM.jpg]

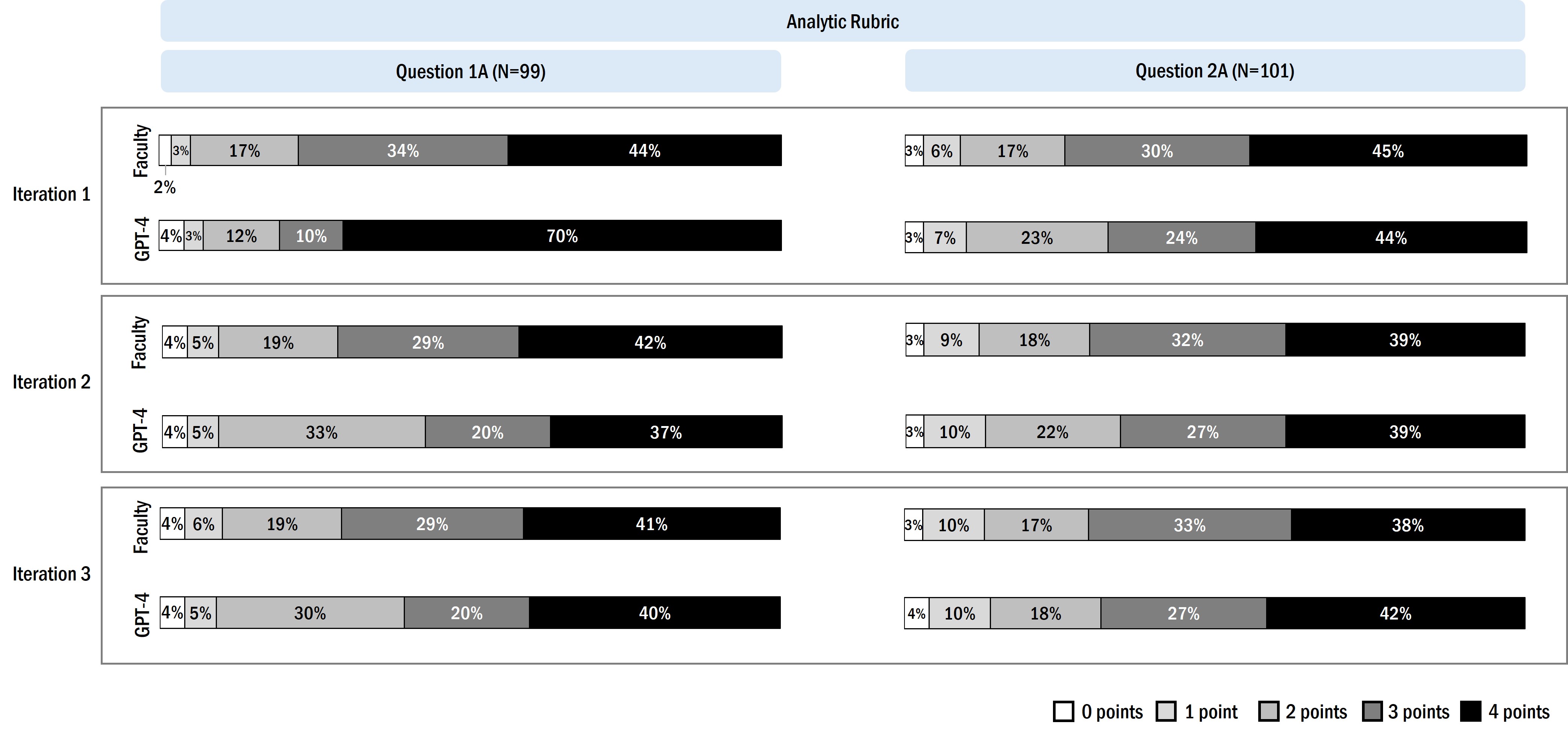

Supplement: Supplementary file 5 — The distribution of scores for questions 1A and 2A for both faculty and GPT-4 for each iteration. (JPG 552 KB) [file 40670_2026_2638_MOESM5_ESM.jpg]

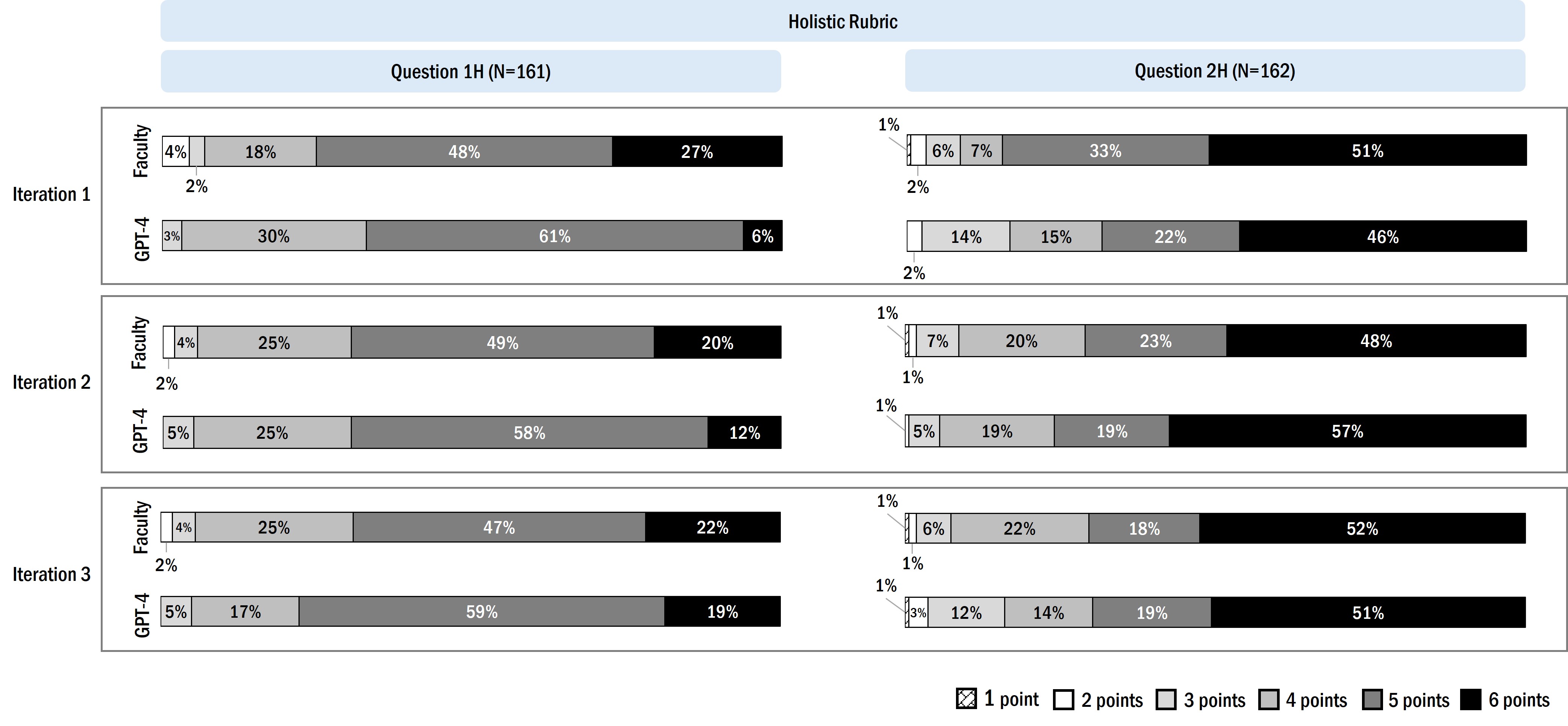

Supplement: Supplementary file 6 — The distribution of scores for questions 1H and 2H for both faculty and GPT-4 for each iteration. (JPG 543 KB) [file 40670_2026_2638_MOESM6_ESM.jpg]
